# Supplementary material for: Patterns of Intron Gain and Loss in Fungi
Source: PLoS Biol. 2004 Nov 30;2(12):e422. doi: 10.1371/journal.pbio.0020422 (PMC532390; doi:10.1371/journal.pbio.0020422)
Supplement: Table S1 — Also available at http://genes.mit.edu/NielsenEtAl/. (4.3 MB ZIP). [file pbio.0020422.st001.zip › NielsenEtAl/html/1183.html]

AN3689.1.NCU06882.1.MG00859.1.FG07044.1


```
 CLUSTAL W (1.82) Multiple Sequence Alignments - Introns Inserted


Sequence 1: NCU06882.1	617 aa
Sequence 2: MG00859.1	515 aa
Sequence 3: FG07044.1	670 aa
Sequence 4: AN3689.1	601 aa
Alignment Length: 684 aa
Number Identitical Residues: 246 aa
Alignment Score (without introns) 12918


MG00859.1 	------------------------~----~--~---------------------------
NCU06882.1	-----------------MGNMPTR2ASLF~RV~G---------------------GISTL
FG07044.1 	MMTETQRHNEIQSHRVPDPTWQPR~LSLF~VL0SPSIKRQRHPLISAPYPQTFFPSLVFF
AN3689.1  	MYLRLVRIQAKKGLKEFNLYLHRK~LATY0HI~RLRG-------VTFTTPPTTRKRKAAL
          	        .  ..             :                  : . . :        

MG00859.1 	--------~-------~----------------------------MDSEDEFMSAISSGD
NCU06882.1	GNYLYSRA1SNLQCNC2TYNCPAS----------------SSATDIDQYDCYPALLGSNP
FG07044.1 	PQYLPSTA~TIPPTTR~KRKAAEATITSANRDEVPNKRVIASAALSDKHALPPTPITTVS
AN3689.1  	AASPTSTT~ATQSCSR~SRAATTTTADSFLDIPDSGSARKRQKKTRKQCPDSAAELTS-T
          	     : : :    .  .  .. ::  :      ...    .    ..     : : :  

MG00859.1 	EDVMSDNE-------------------------------------~-----DMSGAED1F
NCU06882.1	NKLDSRRHSQVSL------LLSHSPIDALPPLPASIIHYGIRVAN1ILPYTDPAIPPD~F
FG07044.1 	NPMDSDEEYMSALSTDDEIMQDDSGDEISAGDGTTFDTPDTPSYP~IMTVTNHRDITD~F
AN3689.1  	NSFNFEREVSESP-------AKVEASEFVTSTSTTMESDDDFMSV~ASSADDFLGTQG~S
          	: .   ..   :         . .  .  .   ::    .        .  :     .  

MG00859.1 	DSE-----~--LDADLDVDLAK-DPPKRKAAHDITFSVYNPDDLRKQQDDLIDEVNMILE
NCU06882.1	DFD-----~--DEPDLDTGNKDSQQAKKHNYLDIPFKVFRPEDIQKQQDGLVDEVNMILD
FG07044.1 	DDEEF---~--DEPDPDFGLAKDVEKKKRAAHVVSYKVYEPSDIQSQQDEMIGEVNMILD
AN3689.1  	DDESLGDD1FGDDFDGGFSKDKDIFSNTRKPYEVDFKVLSPEDIEREQNLQINEVSSILG
          	* :. ...  . : * . .  ..   : :    : :.*  *.*:. :*:  :.**. ** 

MG00859.1 	MRKEDAAILLRHFGWNRERLIEEYMDNRKKVLEVAGLGPNVGGGPQLQVIPGFCCEICCE
NCU06882.1	ISKEEAAILLRHFRWNRERLIEDYMDKPRQVLDAAGLAQTAADKPRLQVIPGFMCDICCE
FG07044.1 	MQKEDAAILLRHFRWNKERLLEDYMDRPEKVMEAAGLSSTTSSSPKLEVIPGFVCDICCE
AN3689.1  	LPPESSAILLRFGRWNREKLIESYMDHPELTLEEAGLGTNFESTPKTEVVPGFTCDICCE
          	:  *.:*****.  **:*:*:*.***. . .:: ***. .  . *: :*:*** *:****

MG00859.1 	DEEGLQSFAMKCGHRYCVDCYRQYLTQKIREEGEAARIQCPSEGCHRIIDAKSLDLLVTP
NCU06882.1	DGDGLESFAIKCGHRYCVDCYRQYLSQKIREEGEAARIQCPADGCNLIIDARSLDILVTP
FG07044.1 	DDDGLESFAMKCGHRYCVDCYRHYLTQKIREEGEAARIQCPSDGCGRILDSASLDVLVTP
AN3689.1  	DGDDLETYAMRCGHRFCVDCYRHYLAQKIREEGEAARIQCPGNDCHMIVDSKSLSLLVTD
          	* :.*:::*::****:******:**:***************.:.*  *:*: **.:*** 

MG00859.1 	ELKER~YHVLLQRTYVEDKDTLKWCPGPDCQNAIDCSIKKKDLHRVVPTVTCNCKMRFCF
NCU06882.1	ELTER~YHELLMRTYVEDKETLKWCPSPDCANAVECGVKKKDLTKVVPTVSCLCGHRFCF
FG07044.1 	ALAGR~YQELLNRTYVEDKDNFKWCPAPDCPNALECGVKKKDLGRIVPTVECRCGFRFCF
AN3689.1  	DLKDR2YQTLLTRTYVDDKENLKWCPAPNCEYAVDCHVKQ--------------------
          	 *  * *: ** ****:**:.:****.*:*  *::* :*:                    

MG00859.1 	GCTLNDHQPAPCELVKMWLKKCADDSETANWISANTKECPKCNSTIE~KNGGCNHMTCRK
NCU06882.1	GCIYTDHQPAPCELVKKWLKKCADDSETANWISANTKECPKCNSTIE~KNGGCNHMTCRK
FG07044.1 	GCPNPDHQPAPCDLVKKWLKKCADDSETANWISANTKECPKCNSTIE~KNGGCNHMTCRK
AN3689.1  	----------------------------------------------Q1KNGGCNHMTCRK
          	                                              : ************

MG00859.1 	CRHEFCWMCMGIWSEHGTSWYNCNRFEEKSGTDARDAQAKSRLSLERYLHYYNRYANHEQ
NCU06882.1	CKYEFCWMCMGLWSEHGTSWYNCNRFEEKSGAEARDAQARSRVSLERYLHYYNRYHNHEQ
FG07044.1 	CKYEFCWMCMGLWSEHGTSWYNCNRYEEKSGSEARDAQAKSRTSLERYLHYYNRYANHEQ
AN3689.1  	CKHEFCWMCMGLWSEHGTSWYNCNRFEEKSGAEARTEQARSRASLERYLHYYNRYANHEQ
          	*::********:*************:*****::**  **:** ************ ****

MG00859.1 	SARLDKDIYHKTEKKMTMLQTASGMSWIEVQYLEAASQALQTCRQTLKWTYAFAFYLSRN
NCU06882.1	SARLDKDLYVKTEKKMVELQKQSGMSWIEVQYLQSASQALQTCRQTLMWTYAFAFYLARN
FG07044.1 	SAKLDKDIAQKTEKKMVQLQSASGMSWIEVQYLNSASQALQTCRQTLKWTYAFAFYLARN
AN3689.1  	SAKLDKDLYLKTEKKMTSLQSQSGLSWIEVQFLDTASQALQQCRQTLKWTYAFAYYLARN
          	**:****:  ******. **. **:******:*::****** ***** ******:**:**

MG00859.1 	HLTTIFEDNQKDLEMAVEALSEMFEKPVAELADSKQKVEIMDKTAYCNRRRVILLEDTAE
NCU06882.1	NLTEIFESNQKDLEMAVENLSEMFEKPVQDLAQPGLKVDIMDKTSYCNKRRVILLEDTAE
FG07044.1 	NLTEIFEDNQKDLEMAVENLSEMFEKPITELSDPKLKVDIMDKTSYCNKRRVILLEDTAE
AN3689.1  	NLTEIFEDNQKDLEMAVESLSEMFEKPVGELAN--LKVDILDKTAYCNKRRVILLSDTAE
          	:** ***.********** ********: :*::   **:*:***:***:******.****

MG00859.1 	NLAN-~------D1IGS------------SASAGRR
NCU06882.1	NLQKG1KWTFNID~LVSGAPIVNPTSTPATTSGSRR
FG07044.1 	NLANG1EWNFNAD~LLAAT---------TSAPVHRR
AN3689.1  	NLKND1LASMLDY~LLKARTDGSLRLIMDILADPGR
          	** :.   .     :  . .  .        .   *
```
